# Atlanta Medical College

**Published:** 1866-09

**Authors:** 


### EDITORIAL AND MISCELLANEOUS.

---

### ATLANTA MEDICAL COLLEGE.

The Regular Session of this Institution for the present year has just been concluded.

The commencement exercises took place at the City Hall on Friday, 31st August. The addresses on the occasion, we must say, were of the most interesting character. We hope to be able to publish one in each of the two succeeding numbers of the Journal.

The graduating Class, numbering twenty-nine, was composed of excellent young men, and, in point of intellect and preparation for the duties of a physician, has not been excelled by any it has been our pleasure to become acquainted with. In fact, the whole Class in attendance upon lectures during the course, was made up of moral, studious and intellectual young men.

The following names compose the Graduating Class:

R. G. Smith, Ga.

Wm. O'Daniel, Ga.

J. H. Russell, Ga.

Wm. D. Sterrett, Texas.

R. E. Baily, Ga.

T. S. Mitchell, Ga.

J. B. Wright, S. C.

J. S. McCants, Ga.

D. C. Bennett, S. C.

C. C. Sanders, Ga.

C. C. Hart, Ga.

W. B. Wells, Ga.

J. A. Hunnicutt, Ga.

W. B. Brantly, Ga.

J. C. Sosnowski, S. C.

H. N. Harris, Ga.

C. L. Williams, Ga.

J. L. M. Hardman, Ga.

Paul Gist, Tenn.

G. B. Atkinson, Ga.

M. Edwards, S. C.

G. F. Wirsen, Sweden.

R. B. Anderson, Ga.

R. S. Cameron, Ga.

M. W. Fowler, Ga.

S. S. Smithwick, Ga.

J. H. Phares, Miss.

W. H. Johnson, Ga.

J. G. Arnall, Ga.

John M. Johnson, M. D., of Georgia, and George F. Jones, M. D., of Tennessee, were admitted *ad eundem gradum* in the Institution.

The announcement for the next Course of Lectures will be issued in a few weeks, in which will be found all desirable information in regard to the operations of the Institution.

In advance of its publication, however, we will mention, for the information of those not familiar with the fees, requisites for graduation, etc., etc., that the next regular Course of Lectures will commence on the first Monday in May 1867.

The usual fees will be required—for the Course of Lectures, $105; Matriculation, $5; Dissecting Ticket, $10. Board in the city, at respectable and well kept boarding-houses, can be had at about $20 per month.

The usual requisites for graduation will be demanded.

At an outlay of several thousand dollars, the College building, apparatus and enclosure have been put in a condition that will allow facilities, equal to those before the war, for instruction in the various branches of Medical Science.

The daily exercises of the Dispensary will, as during the session for the present year, be had every morning before the regular hours for lectures. Clinical medicine, therefore, will receive daily attention, and afford a good variety of interesting cases to be examined and prescribed for in presence of the Class.

